# Synthetic lethality of glutaminolysis inhibition, autophagy inactivation and asparagine depletion in colon cancer

**DOI:** 10.18632/oncotarget.16844

**Published:** 2017-04-05

**Authors:** Jiaqiu Li, Ping Song, Liyuan Zhu, Neelum Aziz, Qiyin Zhou, Yulong Zhang, Wenxia Xu, Lifeng Feng, Dingwei Chen, Xian Wang, Hongchuan Jin

**Affiliations:** ^1^ Department of Medical Oncology, Sir Runrun Shaw Hospital, Medical School of Zhejiang University, Hangzhou, China; ^2^ Laboratory of Cancer Biology, Key Lab of Biotherapy in Zhejiang, Sir Runrun Shaw Hospital, Medical School of Zhejiang University, Hangzhou, China; ^3^ Department of Surgery, Sir Runrun Shaw Hospital, Medical School of Zhejiang University, Hangzhou, China

**Keywords:** colorectal cancer, glutaminolysis, glutamine, synthetic lethality

## Abstract

Cancer cells reprogram metabolism to coordinate their rapid growth. They addict on glutamine metabolism for adenosine triphosphate generation and macromolecule biosynthesis. In this study, we report that glutamine deprivation retarded cell growth and induced prosurvival autophagy. Autophagy inhibition by chloroquine significantly enhanced glutamine starvation induced growth inhibition and apoptosis activation. Asparagine deprivation by L-asparaginase exacerbated growth inhibition induced by glutamine starvation and autophagy blockage. Similar to glutamine starvation, inhibition of glutamine metabolism with a chemical inhibitor currently under clinical evaluation was synthetically lethal with chloroquine and L-asparaginase, drugs approved for the treatment of malaria and leukemia, respectively. In conclusion, inhibiting glutaminolysis was synthetically lethal with autophagy inhibition and asparagine depletion. Therefore, targeting glutaminolysis could be a promising approach for colorectal cancer treatment.

## INTRODUCTION

Colorectal cancer (CRC) is the third most common cancer in men and the second in women worldwide [1]. Despite great advance especially in early detection with endoscopy examination, the lack of effective therapeutic approaches contributed to high rates of recurrence and morbidity even after surgical resection [2, 3]. Targeted therapy has been recognized as a new choice for cancer treatment. However, most of targeted therapies such as antibodies against receptor tyrosine kinases are based on targeting signaling molecules [4, 5]. Meanwhile, metabolism has to be reprogrammed to coordinate the activation of oncogenic signaling during malignant transformation. Therefore, targeting reprogrammed metabolism in cancer cells was recently proposed to be a promising therapeutic strategy [6].

Cancer cells have to consume more nutrients, mainly glucose and glutamine, to fulfill the increased proliferation demands for energy generation and macromolecule biosynthesis. Early in the beginning of last century, Germany biochemist Otto Warburg first described a significantly increased uptake of glucose by cancer cells [7]. Although positron emission tomography (PET) using glucose analogues as tumor tracer has been applied successfully in the clinic, little effects of targeting glycolysis on cancer treatment have been found [8, 9]. In addition to elevated aerobic glycolysis, cancer cells depend more on glutaminolysis [10, 11]. As a donor for both carbon and nitrogen, glutamine can be metabolized in tricarboxylic acid cycle (TCA) cycle to generate energy and produce intermediates for the synthesis of macromolecules such as protein, lipid and nucleotide [12, 13]. Tumor cells produce less adenosine triphosphate (ATP) from glycolysis and become addicted on glutaminolysis as TCA cycle anapleurosis [14, 15]. Such addictions on glutamine metabolism could be stimulated by well-known oncoproteins such as c-myc and k-Ras, further highlighting the relevance of glutamine metabolism to human carcinogenesis [16, 17]. As a fact, glutamine tracers have succeeded to evaluate tumor formation in brain, where glucose was heavily consumed in normal tissues [18]. Targeting glutamine metabolism for cancer treatment was also under active investigations.

Many studies have described the association of autophagy with glutamine metabolism [19, 20]. Under environmental stresses, autophagy can be activated to degrade nonessential macromolecules to help cancer cells survive from the stressful conditions [21]. Therefore, autophagy is critical for not only the maintenance of the intracellular homeostasis but also the cellular response to metabolic stress. In this study, we found that inhibition of glutamine metabolism indeed could activate compensatory responses such as prosurvival autophagy in colorectal cancer cells. Glutamine starvation or inhibition of glutamine metabolism with chemicals under clinical evaluation was synthetically lethal with the combination of autophagy inhibition and extracellular asparagine depletion. Hence, targeting glutamine metabolism could be a promising approach for the treatment of human CRC.

## RESULTS

### Glutamine starvation activates autophagy in colorectal cancer cells

**Figure 1:**
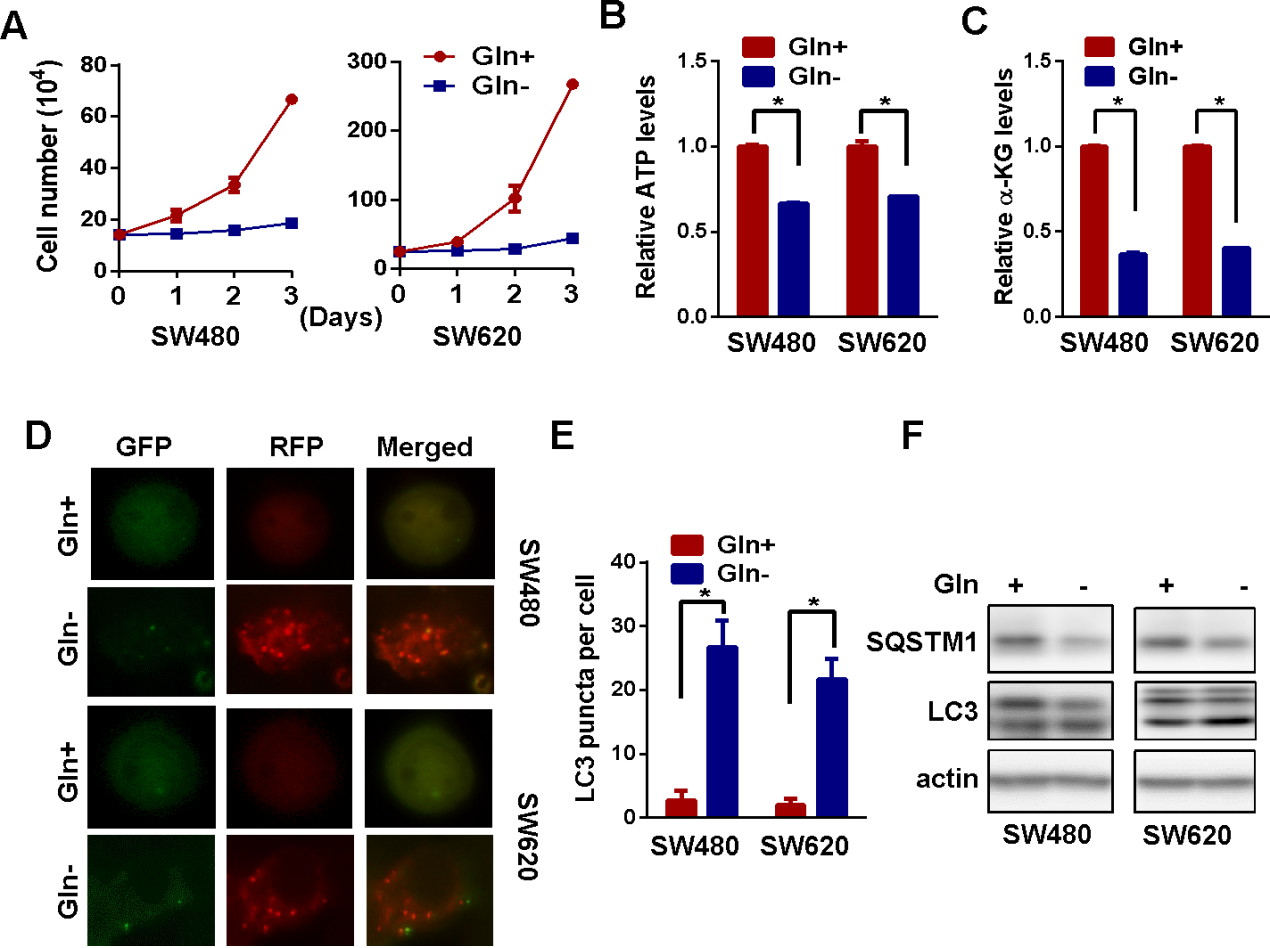
Glutamine starvation activates autophagy **(A)** Cell growth in the presence or absence of glutamine was determined by cell counting for three days. Two-way ANOVA test was used to determine the statistical difference (p < 0.01 for SW480 and 620 cells). **(B)** and **(C)** The intracellular level of ATP **(B)** and α-KG **(C)** in the presence or absence of glutamine for 72h was detected by metabolic analyses. The asterisks indicate statistical significance (p < 0.05). **(D)** and **(E)** mRFP-GFP-LC3 distribution in SW480 and SW620 cells with or without glutamine deprivation for was analyzed by confocal microscopy. Red LC3 puncta per cell in are shown as mean ± SD in the right panel. **(F)** The expression of LC3 and SQSTM1 before and after glutamine deprivation for 24 h (for LC3 detection) or 72 h (for SQSTM1 detection) were explored by western blotting.

In order to figure out the relevance of glutamine to CRC, we firstly removed glutamine from the medium *in vitro* cultured colorectal carcinoma cells. We found that glutamine deprivation significantly reduced the cell growth and viability of SW480 and SW620 cells (Figure 1A and Supplementary Figure 1). Meanwhile, the intracellular level of ATP and alpha-ketoglutarate (alpha-KG) was also decreased (Figure 1B and 1C), confirming the importance of glutamine to energy metabolism and cellular viability of CRC cells. It has been reported that autophagy could be activated in response to amino acid deprivation [22]. Indeed, we found light chain 3 beta (LC3) puncta in acidic vesicles, a well-known indicator of autophagy activation [23], were significantly increased in SW480 and SW620 cells after glutamine starvation (Figure 1D and 1E). Rather than an increase in the number of yellow vesicles in cells transfected with GFP-RFP-LC3, the increase in the number of red vesicles indicated enhanced turnover from autophagosome to autolysosome after glutamine deprivation. Consistently, the conversion of LC3-l to LC3-ll was increased while sequestosome 1 (SQSTM1) expression decreased in both SW480 and SW620 cells after glutamine deprivation, confirming the activation of autophagy in response to glutamine starvation (Figure 1F).

### Inactivation of autophagy enhanced growth inhibition induced by glutamine starvation

**Figure 2:**
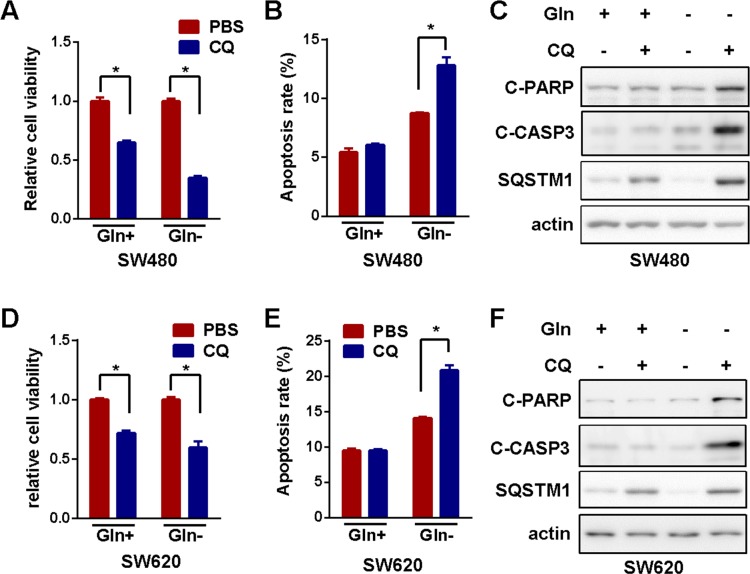
Inactivation of autophagy enhanced growth inhibition induced by glutamine starvation **(A)** The effect of autophagy inhibitor-CQ (10μM) on cell viability in the presence or absence of glutamine for 72h was determined by MTS assay. **(B)** and **(C)** The effect of autophagy inhibitor-CQ (10μM) on cell apoptosis in the presence or absence of glutamine for 72h was assessed using flow cytometry **(B)** and Western blotting detection of cleaved caspase 3 and PARP cleavage (for 48h) **(C)**. **(D)** MTS assay in SW620 cells as in **(A)**. **(E)** and **(F)** Apoptosis detection in SW620 cells as in **(B)** and **(C)**, respectively. All experiments were repeated for 3 times and the representative results were shown. The asterisks indicate statistical difference (p < 0.05).

Presumably, autophagy was activated to promote survival under various stresses such as amino acid shortage [24]. However, autophagy was also reported to promote cell death [25]. In order to clarify the relevance of autophagy induced by glutamine starvation in colorectal cancer cells, we blocked autophagy with clinical available autophagy inhibitor, chloroquine (CQ), in colorectal cancer cells before and after glutamine starvation. The inhibition of autophagy by CQ significantly potentiated viability inhibition (Figure 2A) and apoptosis activation (Figure 2B and 2C) induced by glutamine deprivation in SW480. Similar results were achieved in SW620 cells (Figure 2D, 2E and 2F). All of these results indicated that glutamine starvation induced prosurvival autophagy in colorectal cancer cells. Therefore, combination of autophagy blockage with glutamine deprivation could be a novel strategy for the treatment of CRC.

### Glutamine starvation is synthetically lethal with autophagy inhibition and asparagine depletion

**Figure 3:**
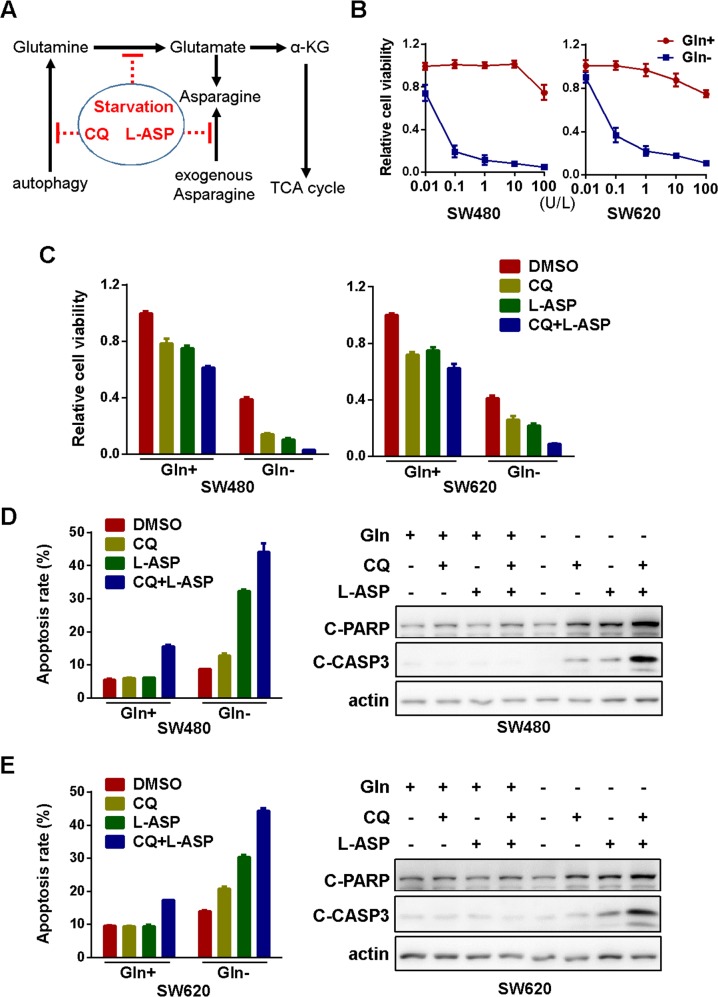
Glutamine starvation is synthetically lethal with autophagy inhibition and asparagine depletion **(A)** Schematic diagram showing the targeting of glutaminolysis in cancer cells. **(B)** The effect of L-asparaginase (L-ASP) on cell viability in the presence or absence of glutamine for 72h was determined by MTS assay. All experiments were performed in triplicate and repeated experiments three times. The most representative results were shown. **(C)** The effect CQ (10μM) and L-ASP (100U/L) on cell viability in the presence or absence of glutamine for 72h was determined by MTS assay (CQ, L-ASP, CQ+L-ASP vs DMSO in both Gln+ and Gln- groups, p < 0.05; CQ+L-ASP in Gln+ vs CQ+L-ASP in Gln-, p < 0.05). **(D)** The effect of CQ (10μM) and L-ASP (100U/L) on apoptosis of SW480 cells in the presence or absence of glutamine for 72h was assessed using flow cytometry (left panel, CQ+L-ASP vs DMSO in Gln+ group, p < 0.05; CQ, L-ASP, CQ+L-ASP vs DMSO in Gln- group, p < 0.05; CQ+L-ASP in Gln+ vs CQ+L-ASP in Gln-, p < 0.05) and Western blotting (for 48h) (right panel). **(E)** The effect of CQ (10μM) and L-ASP (100U/L) on apoptosis of SW620 cells in the presence or absence of glutamine for 72h was assessed using flow cytometry (left panel, CQ+L-ASP vs DMSO in Gln+ groups, p < 0.05; CQ, L-ASP, CQ+L-ASP vs DMSO in Gln- groups, p < 0.05; CQ+L-ASP in Gln+ vs CQ+L-ASP in Gln-, p < 0.05) and Western blotting (for 48h) (right panel).

Other than carbon donor for ATP generation in TCA cycle, glutamine is also the important nitrogen source for the synthesis of other amino acids or nucleotides in mammalian cells [26]. As one of the metabolic products of glutamine in this process, asparagine is essential for protein biosynthesis and cellular survival. While lymphoblastic leukemia cells lack the ability to synthesize asparagine, solid tumor cells could either produce asparagine by themselves or import asparagine from extracellular environment. Therefore, depletion of extracellular asparagine by anti-leukemia drug L-asparaginase (L-ASP) has negligible effects on solid tumors [27]. However, tumor cells could become susceptible to asparagine deprivation once its biosynthesis from glutamine was inhibited (Figure 3A). Indeed, in the absence of glutamine, L-ASP significant inhibited the viability of both SW480 and SW620 cells, with the IC50 value lower than 0.1U/L (Figure 3B). Furthermore, the blockage of compensative glutamine production by autophagy inhibition further exacerbated viability inhibition (Figure 3C) and apoptosis induction (SW480 in Figure 3D and SW620 in Figure 3E and Supplementary Figure 4). All of these data suggested that the inhibition of glutamine catabolism is synthetically lethal with autophagy blockage and asparagine depletion.

### Pharmaceutical inhibition of glutaminolysis in combination of autophagy inhibition and asparagine depletion

**Figure 4:**
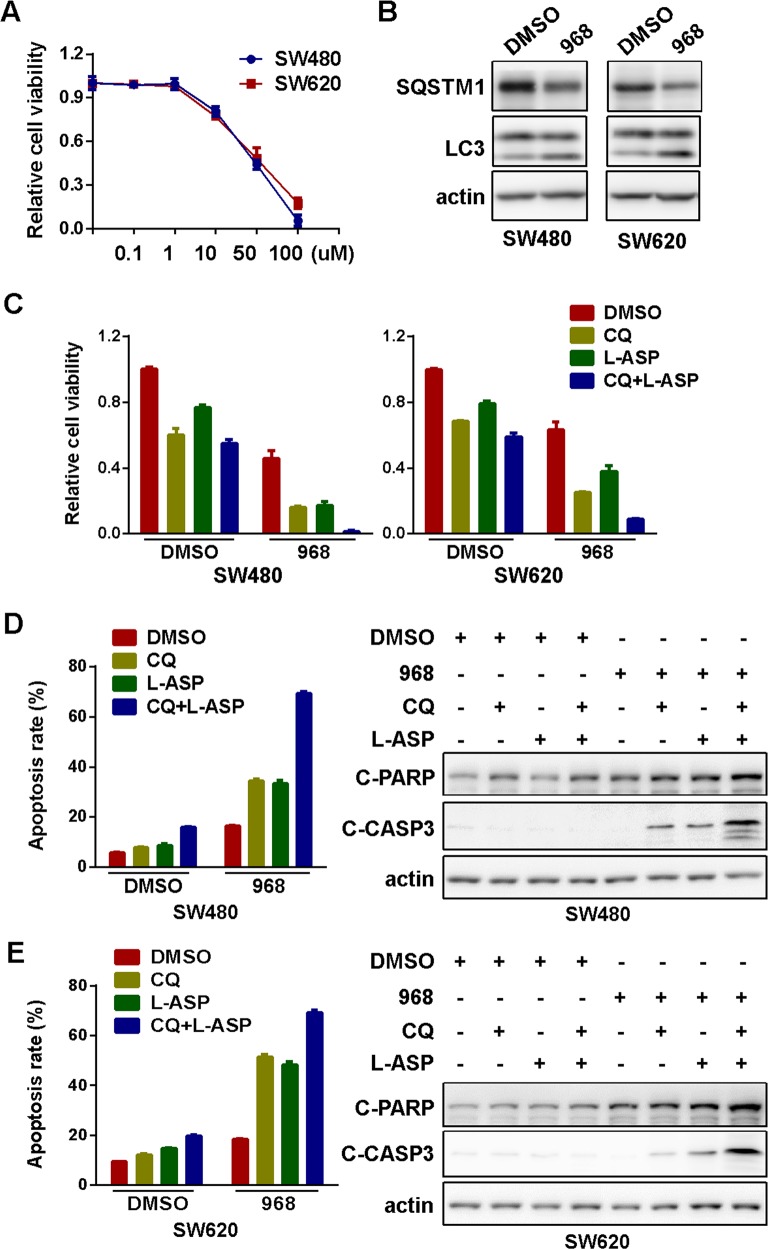
Pharmaceutical inhibition of glutaminolysis in combination of autophagy inhibition and asparagine depletion inhibited cell growth **(A)** The effect of glutaminolysis inhibitor-compound 968 (968) on viability of SW480 and SW620 cell for 72h were explored by MTS assay. All experiments were performed in triplicate and repeated experiments three times. The most representative results were shown. **(B)** The effect of compound 968 (20μM) on the level of LC3, SQSTM1 for 72h were explored by Western blotting. **(C)** The effect of CQ (10μM) and L-ASP (100U/L) on cell viability before and after 968 treatment (20μM) were determined by MTS assay (CQ, L-ASP, CQ+L-ASP vs DMSO in both DMSO and 968 groups, p < 0.05; CQ+L-ASP in DMSO vs CQ+L-ASP in 968, p < 0.05). **(D)** The effect of CQ (10μM) and L-ASP (100U/L) on apoptosis of SW480 cells before and after glutaminolysis inhibition for 72h were assessed using flow cytometry (left panel, CQ+L-ASP vs DMSO in DMSO group, p < 0.05; CQ, L-ASP, CQ+L-ASP vs DMSO in 968 group, p < 0.05; CQ+L-ASP in DMSO vs CQ+L-ASP in 968, p < 0.05) and Western blotting (for 48h) (right panel). **(E)** The effect of CQ (10μM) and L-ASP (100U/L) on apoptosis of SW620 cells before and after glutaminolysis inhibition for 72h were assessed using flow cytometry (left panel, CQ+L-ASP vs DMSO in DMSO group, p < 0.05; CQ, L-ASP, CQ+L-ASP vs DMSO in 968 group, p < 0.05; CQ+L-ASP in DMSO vs CQ+L-ASP in 968, p < 0.05) and Western blotting (for 48h) (right panel).

We next explore the possibility of pharmaceutical inhibition of glutaminolysis for CRC treatment. We first compared the inhibition of CRC cell viability by bis-2-(5-phenylacetamido-1,2,4-thiadiazol-2-yl) ethyl sulfide (BPTES), compound 968 and CB-839, three commercially available chemical inhibitors of glutaminolysis (Supplementary Figure 2). We chose compound 968 for further investigations since it seemed to be the most potent to inhibit cellular viability in colorectal cancer cells. Moreover, colorectal cancer cells were more sensitive to compound 968 than non-cancer cell line HEK293T (Supplementary Figure 3). Compound 968 inhibited the viability of SW480 and SW620 cells in a dose-dependent manner after incubation for 72h (Figure 4A) [28, 29]. Similar to glutamine starvation, glutaminolysis inhibition by compound 968 effectively induced autophagy in both SW480 and SW620 cells (Figure 4B). Likewise, compound 968 exerted a significant synergy with CQ and L-ASP to inhibit viability (Figure 4C) and induce apoptosis (Figure 4D and 4E and Supplementary Figure 5) in CRC cells. Overall, the chemical inhibitor of glutaminolysis phenocopied glutamine restriction to confer synthetic lethality with autophagy inhibition and asparagine depletion, providing a possibility of glutaminolysis inhibitor-including combination regiments for the treatment of CRC.

## DISCUSSION

By blocking specific molecules essential for tumor cells, targeted therapy was expected to be more effective and safer than older forms of treatments such as cytotoxic chemotherapy. Most of such therapeutic targets are mainly amplified or mutated oncogenes playing critical roles in mitogenic signaling pathways. However, in addition to oncogenic signaling pathways, cellular machinery regulating cancer cell metabolism contains numerous potential targets since metabolism is reprogrammed to accommodate malignant transformation [6]. In this study, we found targeted inhibition of glutamine metabolism suppressed colorectal carcinogenesis especially when combined with autophagy inhibition and extracellular asparagine depletion, thus representing a promising approach for the treatment of human CRC.

As a compensation to elevated aerobic glycolysis, cancer cells consume more glutamine for energy generation and TCA cycle anapleurosis [10, 11]. Despite glucose analogues have been applied as tumor tracers successfully, little effects of targeting metabolism on cancer treatment have been reported [8, 9]. This could be attributed to the dynamic remodeling of metabolism to enable cellular survival under unfavorable conditions. For example, upon nutrient restriction, a self-digestion process called autophagy can be activated to alternatively provide amino acids and other materials essential for cellular survival and growth [30–34]. A recent manuscript by Seo JW and his colleague identified the key role for autophagy in glutamine metabolism in pancreatic ductal adenocarcinoma cells [35]. Glutamine deprivation rather than glucose shortage induced the formation of macropinocytosis-associated autophagy by the translocation of transcription factor EB (TFEB) into the nucleus. Therefore, inhibiting the activation of prosurvival autophagy could synergize metabolism inhibition in anti-cancer treatment. In our study, we first removed glutamine from cultured medium in colorectal cancer cells SW480, SW620, HCT116 and HT29. Then we selected SW480 and SW620 which were more sensitive than other cells after glutamine deprivation (data not shown). We found glutamine deprivation indeed could induce autophagy formation by the conversion LC3 I to LC3 II which means the connection of LC3 with autophagosome membrane, and p62 degradation in these colorectal cancer cells. Moreover, we demonstrated that the combination of autophagy inhibitor CQ significantly potentiated inhibitory effect of glutamine starvation or glutaminolysis targeting, which further identifying the significance of glutamine to colorectal cancer cells.

**Figure 5:**
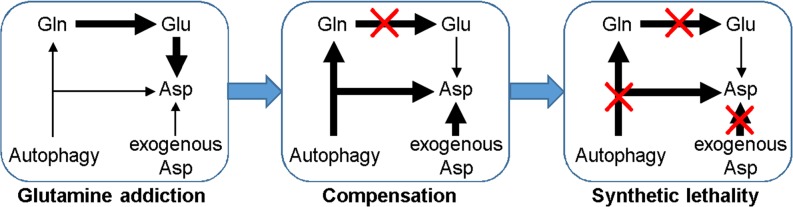
Cancer cells addict on increased glutamine metabolism to sustain ATP generation and biosynthesis essential for cellular survival and proliferation Inhibition of glutaminolysis will activate compensatory responses such as prosurvival autophagy and consuming exogenous rather than glutamine-derived asparagine. Thus, it would be necessary to combine glutaminolysis targeting with autophagy inhibition and asparagine depletion to kill cancer cells.

In addition, glutamine is indispensable for cancer cell growth not only for energy supply but also for macromolecular biosynthesis. For example, glutamine metabolism can produce asparagine in solid tumor cells but not leukemia cells, conferring intrinsic resistance to anti-leukemia drug L-asparaginase (L-ASP) [27]. However, tumor cells could become extremely susceptible to asparagine deprivation once its biosynthesis from glutamine was inhibited. In both SW480 and SW620 cells under glutamine starvation, the IC50 value of L-ASP decreased to less than 0.1U/L which is even 100-1000 times less than the IC50 value of L-ASP in leukemia cells [36]. The combination of glutaminolysis inhibition with CQ and L-ASP is synthetically lethal to greatly inhibit *in vitro* growth of colorectal cancer cells, representing a promising strategy for the intervention of colorectal cancer (Figure 5). Importantly, both CQ and L-ASP have been used clinically. Therefore, it would be interesting to know the clinical efficacy of glutaminolysis inhibitors in combination with L-ASP and CQ for the treatment of CRC.

The glutamine analogue, 6-diazo-5-oxo-L-norleucine (DON) showed an inhibitory effect against various tumors in nude mice [37]. However, due to its weaker selectivity and severe toxicity, DON was abandoned for further clinical development [38, 39]. More recently, a group of new glutaminolysis inhibitors including BPTES, compound 968 and CB-839 have been identified [29, 40, 41]. While BPTES and CB-839 are similar in structure, the IC50 value of BPTES for glutaminase inhibition was much lower. Several phase I clinical trials are ongoing to evaluate the safety, pharmacokinetics and pharmacodynamics of CB-839 in patients with hematological malignancies and solid tumors such as breast cancer, renal cancer and lung cancer (www.clinicaltrials.gov). No adverse side effects has been reported. Although compound 968 has a distinct structure, colorectal cancer cells were more sensitive to compound 968 than BPTES and CB-839 (Supplementary Figure 2). Of course, compound 968 also have certain inhibitory effects on non-tumor human cells such as HEK293T. However, CRC cells are more sensitive to compound 968 than HEK293T cells (Supplementary Figure 3). Nevertheless, the safety and clinical efficacy of compound 968 for targeting glutamine metabolism in human cancers need to be evaluated in further clinical trials.

In summary, inhibition of glutamine metabolism was synthetically lethal with the combination of autophagy inhibition and extracellular asparagine depletion. Therefore, targeting impair glutamine metabolism could be a promising approach for the management of human CRC.

## MATERIALS AND METHODS

### Cell, antibodies and chemicals

Human colorectal cancer cell lines were purchased from the American Type Culture Collection (ATCC). Cells were cultured in RPMI-1640 (Invitrogen, 11875-093) or DMEM (Invitrogen, 11965-092) medium supplemented with 10% fetal bovine serum (FBS). All cells were incubated at 37°C with 5% CO2 and 95% humidity. The following antibodies were used: cleaved caspase 3 (9661-s, CST), cleaved PARP (9541-s, CST), LC3B (Nb100-2220, NOVUS), SQSTM1 (pm045, MBL), β-actin (4970L, CST). Compound 968 was purchased from Merck Millipore (352010). Chloroquine (C6628) was obtained from Sigma while L-ASP was from Slpharm.

### Immunoblotting

The cells treated as indicated for 48h or 72h were washed once by PBS and lysed by RIPA buffer. After protein quantification, the lysates were separated by SDS-PAGE and transferred to polyvinylidene fluoride (PVDF) membranes for primary antibody incubation overnight at 4°C after being blocked with 5% milk in tris buffered saline with Tween (TBST). Then the membranes were washed by TBST for three times and then incubated with the secondary antibody conjugated with horseradish peroxidase (HRP) (1:5000,111-035-003, Jackson Immuno Research, USA) at 37°C for 2 h. Finally, the membranes were visualized with enhanced chemiluminescence (EMD Millipore, 17-373SP).

### Cell viability assay

6000 cells seeded overnight in 96-well plates were treated as indicated. The cell viability were measured after treatment with different drugs for 72h by The CellTiter 96 AQueous Non-Radioactive Cell Proliferation Assay Kit (Promega). The absorbance value of each well was measured by a microplate reader at 490 nm. Each experiment was repeated for three times.

### Apoptosis detection

Cell apoptosis was detected by flow cytometer analysis and Western blotting. For flow cytometer analysis of apoptosis, cells treated as indicated for 72h were harvested by trypsin and re-suspended in 100 ul 1 x binding buffer. 5 ul FITC Annexin V and PI (556547, BD Biosciences, USA) was added to the cell suspension and then incubated for 15 min at room temperature. After dilution with 400 ul binding buffer, the samples were analyzed by ACS Calibur flow cytometer (BD). For apoptosis by Western blotting, cleaved caspase 3 and cleavage of PARP (poly ADP-ribose polymerase) was analyzed by the antibody specifically recognize cleaved PARP (9661-s and 9541-s, CST).

### Tandem mRFP-GFP-LC3 fluorescence microscopy

Cells were transfected with a plasmid containing mRFP-GFP-LC3 gene for 24h. Then the transfected cells were reseeded on glass coverslips in 6-well plates and cultured overnight. Subsequently, the cells were treated with culture medium with glutamine or not for 24h. The treated cells were fixed for 10 min by 4% formaldehyde and washed by PBS. The coverslips were transferred to glass slides and stained with diamidino-phenyl-indole (DAPI). The GFP/RFP signals were acquired by a Zeiss LSM 710 confocal microscope system (Carl Zeiss, Germany). Three different cells in SW480 and SW620 was counted the LC3 puncta.

### Metabolites analyses

Cells were seeded in 6-well plates for overnight. After the indicated treatments for 72h, the concentrations of glutamine, ATP or α-KG was measured by Glutamine Assay Kit (Abnova, KA1627), ATP Assay Kit (Beyotime, S0026) or α-Ketoglutarate Colorimetric/Fluorometric Assay Kit (BioVision, K677-100), based on the protocols provided in the kit.

### Statistical analysis

All data were expressed as mean ± SD. Unless specifically stated, Student's t-test was performed for statistical significance analysis. P value < 0.05 was considered as statistically significant.

## SUPPLEMENTARY MATERIALS AND FIGURES



## Abbreviations

alpha-KG: alpha-ketoglutarate
ATP: adenosine triphosphate
ATCC: American Type Culture Collection
BPTES: bis-2-(5-phenylacetamido-1,2,4-thiadiazol-2-yl)ethyl sulfide
CQ: chloroquine
CRC: colorectal cancer
DAPI: diamidino-phenyl-indole
DON: 6-diazo-5-oxo-L-norleucine
FBS: fetal bovine serum
HRP: horseradish peroxidase
GFP: green fluorescent protein
L-ASP: L-asparaginase
LC3: light chain 3 beta
PET: positron emission tomography
PVDF: poly vinyl dene fluoride
RFP: red fluorescent protein
SQSTM1: sequestosome 1
TCA: tricarboxylic acid cycle
TBST: tris buffered saline with Tween
