# Supplementary material for: Synthetic lethality of glutaminolysis inhibition, autophagy inactivation and asparagine depletion in colon cancer
Source: Oncotarget. 2017 Apr 5;8(26):42664–72. doi: 10.18632/oncotarget.16844 (PMC5522096; doi:10.18632/oncotarget.16844)
Supplement: Supplementary file 1 [file oncotarget-08-42664-s001.pdf]

Synthetic lethality of glutaminolysis inhibition, autophagy inactivation and asparagine depletion in colon cancer

SUPPLEMENTARY MATERIALS

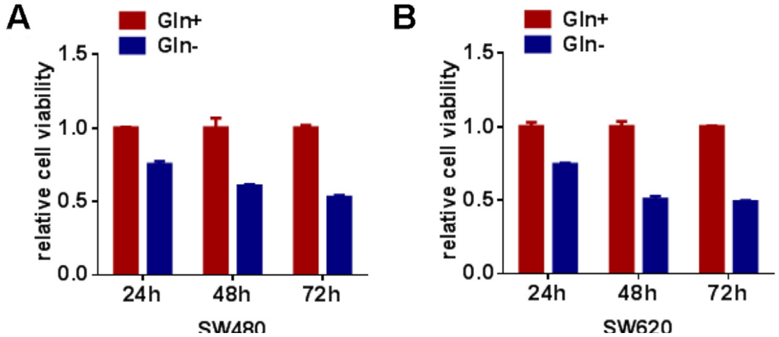

Supplementary Figure 1: The effect of glutamine deprivation on cell viability were analyzed by MTS assay.

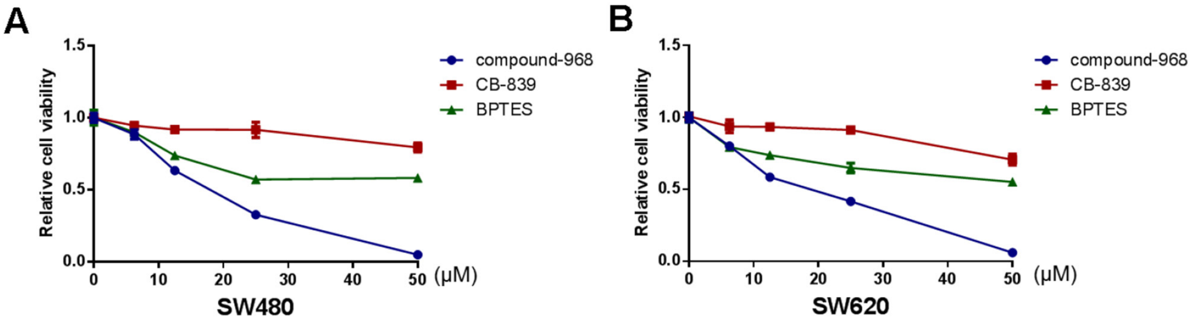

Supplementary Figure 2: The effect of different GLS1 inhibitors on cell viability were analyzed by MTS assay.

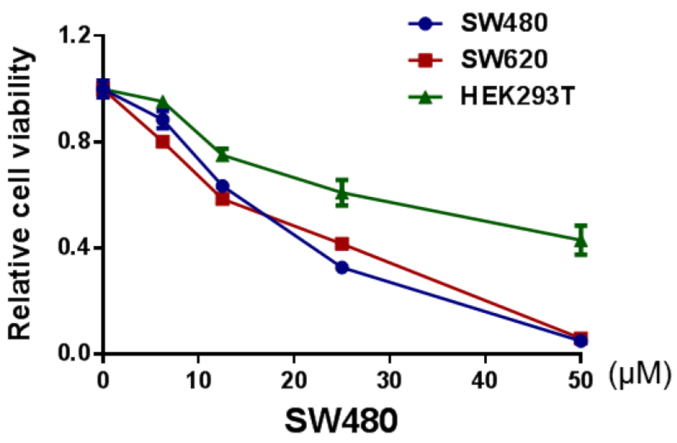

Supplementary Figure 3: The effect of compound 968 on viability of cells as indicated were analyzed by MTS assay.

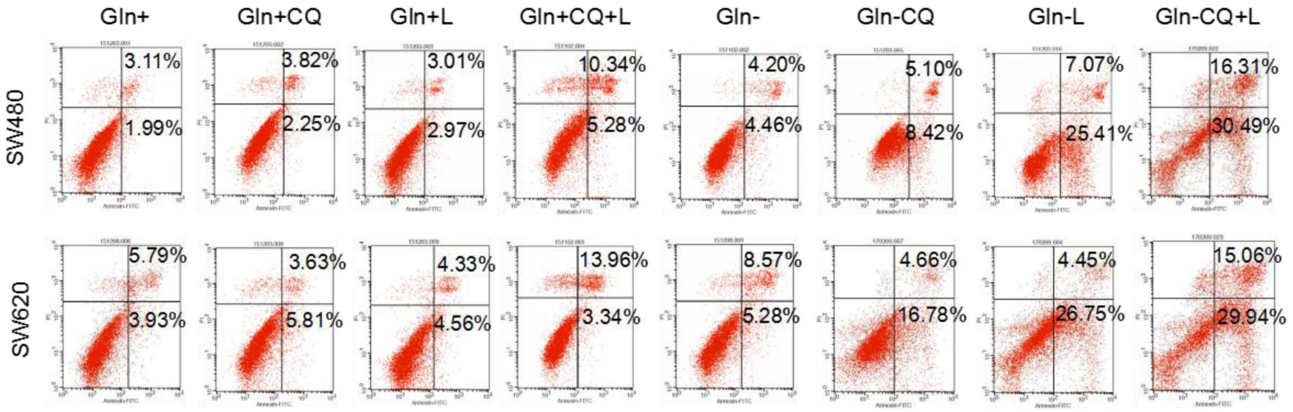

Supplementary Figure 4: The effect of CQ (10uM) and L-ASP (100U/L) on apoptosis of SW480 and SW620 cells in the presence or absence of glutamine for 72h was assessed using flow cytometry. Apoptotic cells were positive for FITC- Annexin V5. This figure is related with Figure 3D and 3E.

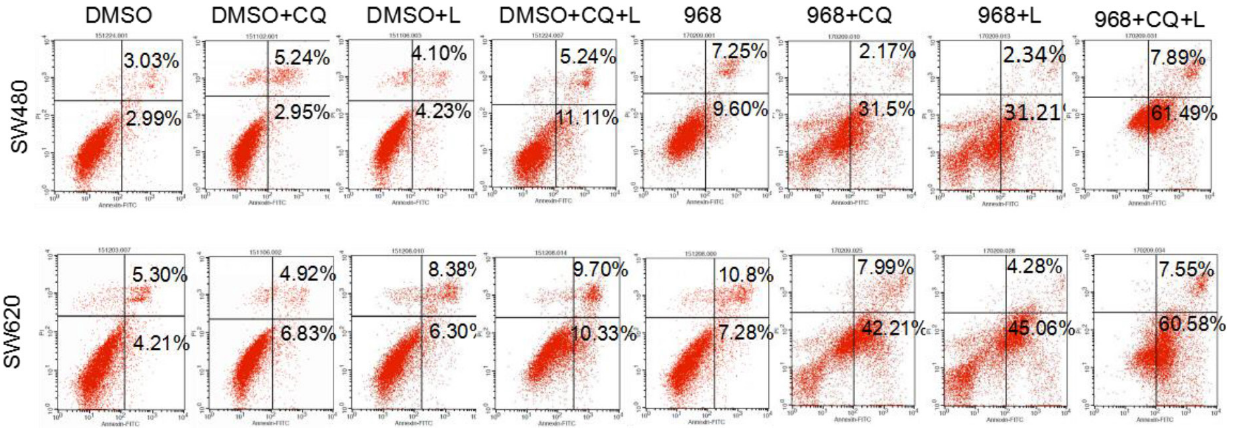

Supplementary Figure 5: The effect of CQ (10uM) and L-ASP (100U/L) on apoptosis of SW480 and SW620 cells before and after glutaminolysis inhibition for 72h was assessed using flow cytometry. Apoptotic cells were positive for FITC-Annexin V5. This figure is related with Figure 4D and 4E.
